# Independent and combined effects of daytime heat stress and night-time recovery determine thermal performance

**DOI:** 10.1242/bio.038141

**Published:** 2019-03-05

**Authors:** Chun-Ming Bai, Gang Ma, Wan-Zhi Cai, Chun-Sen Ma

**Affiliations:** 1 Climate Change Biology Research Group, State Key Laboratory for Biology of Plant Diseases and Insect Pests, Institute of Plant Protection, Chinese Academy of Agricultural Sciences, No. 2, Yuanmingyuan West Road, Haidian District, Beijing 100193, China; 2 Department of Entomology, College of Plant Protection, China Agricultural University, No. 2, Yuanmingyuan West Road, Haidian District, Beijing 100193, China

**Keywords:** Asymmetric warming, Climate change, Critical thermal maximum, Heat tolerance, *Propylea japonica*, Temperature variability

## Abstract

Organisms often experience adverse high temperatures during the daytime, but they may also recover or repair themselves during the night-time when temperatures are more moderate. Thermal effects of daily fluctuating temperatures may thus be divided into two opposite processes (i.e. negative effects of daytime heat stress and positive effects of night-time recovery). Despite recent progress on the consequences of increased daily temperature variability, the independent and combined effects of daytime and night-time temperatures on organism performance remain unclear. By independently manipulating daily maximum and minimum temperatures, we tested how changes in daytime heat stress and night-time recovery affect development, survival and heat tolerance of the lady beetle species *Propylea japonica*. Thermal effects on development and survival differed between daytime and night-time. Daytime high temperatures had negative effects whereas night-time mild temperatures had positive effects. The extent of daytime heat stress and night-time recovery also affected development and critical thermal maximum, which indicates that there were both independent and combined effects of daytime and night-time temperatures on thermal performances. Our findings provide insight into the thermal effect of day-to-night temperature variability and have important implications for predicting the impacts of diel asymmetric warming under climate change.

## Abstract (precis)

**Summary:** Daytime and night-time temperatures that organisms experience in fluctuating environments can determine thermal performance and have implications for predicting the impacts of diel asymmetric warming under climate change.

## INTRODUCTION

Climate warming leads to a substantial increase in global mean temperatures as well as daily maximum and minimum temperatures worldwide (Karl et al., 1993; Easterling et al., 1997; IPCC, 2013), and it has resulted in significant impacts on species of many taxa (Easterling et al., 2000; Parmesan et al., 2000; Jiguet et al., 2011; Peng et al., 2013; Sørensen et al., 2016; Barton and Schmitz, 2018). Most previous studies concerning effects of temperature change are conducted under constant conditions (Easterling et al., 2000; Smith, 2011; Lloret et al., 2012; Reyer et al., 2013). However, ambient temperatures vary and most organisms experience fluctuating thermal environments in nature (Fischer et al., 2011; Colinet et al., 2015). Importantly, the effects of constant and fluctuating temperatures can be very different (Bozinovic et al., 2013; Zhao et al., 2014; Ma et al., 2015a). Compared to fluctuating temperatures, constant temperatures often overestimated or underestimated thermal effects on organismal performances, such as metabolism, locomotion, development, survival, reproduction and population growth (Du and Ji, 2006; Ragland and Kingsolver, 2008; Estay et al., 2014; Zhao et al., 2014; Ma et al., 2015a,b; Kingsolver et al., 2016). Thus, to mimic the effects of natural fluctuating temperatures, daily temperature fluctuations have received increased attention in recent years (Zhao et al., 2014; Ma et al., 2015a; Bozinovic et al., 2016).

Nevertheless, previous research concerning the effect of fluctuating temperatures focused mainly on either (1) changes in amplitudes of daily temperature variability with the same means (Lambrechts et al., 2011; Lyons et al., 2013; Xing et al., 2014) or (2) shifts in temperature means with the same variances (Paaijmans et al., 2010; 2013). In these studies, daily maximum and minimum temperatures were manipulated to shift concurrently in the same or opposite direction. That experimental design may not allow us to differentiate the effects of changes in temperature maxima from that in minima. However, changes in daily maximum or minimum temperatures can alter thermal performance curves and influence key fitness components including development, survival, fecundity and longevity (Zhao et al., 2014; Ma et al., 2015a,b).

Thermal performance curves show that increases in temperatures below optima have positive effects on organisms whereas increases in temperatures above optima have negative effects (Angilletta, 2009; Bozinovic et al., 2011; Speights et al., 2017). Terrestrial invertebrates often experience adverse thermal environments caused by daytime extreme high temperatures in summer days, especially in the context of ongoing climate warming (Clusella-Trullas et al., 2011; Gillespie et al., 2012; Sentis et al., 2013; Ma et al., 2015a,b; Kingsolver et al., 2016). Meanwhile, they are also expected to recover or repair themselves during the cooler intervals in between repeated exposure to high temperatures (Bozinovic et al., 2011; Colinet et al., 2015; Ma et al., 2015a; Speights et al., 2017). Hence, thermal effects of daily fluctuating temperatures may be divided into two biological processes (i.e. negative effects of daytime heat stress and positive effects of night-time recovery). So far, however, we still know little about the thermal effects of daytime heat stress and night-time recovery on organism performance.

Here we used a lady beetle *Propylea japonica*, a predatory insect species, as our model organism. First, we measured egg development rate and survival, key fitness components, under different combinations of daily temperature maxima and minima to differentiate the thermal effects of changes in daytime heat stress from those in night-time recovery. Then, we measured the critical thermal maximum (CT_max_) of first-instar larva that had newly hatched either in the morning or in the evening in an effort to understand how changes in daytime heat stress and/or night-time recovery affect organism thermal performance.

## RESULTS

### Development

**Fig. 1.**
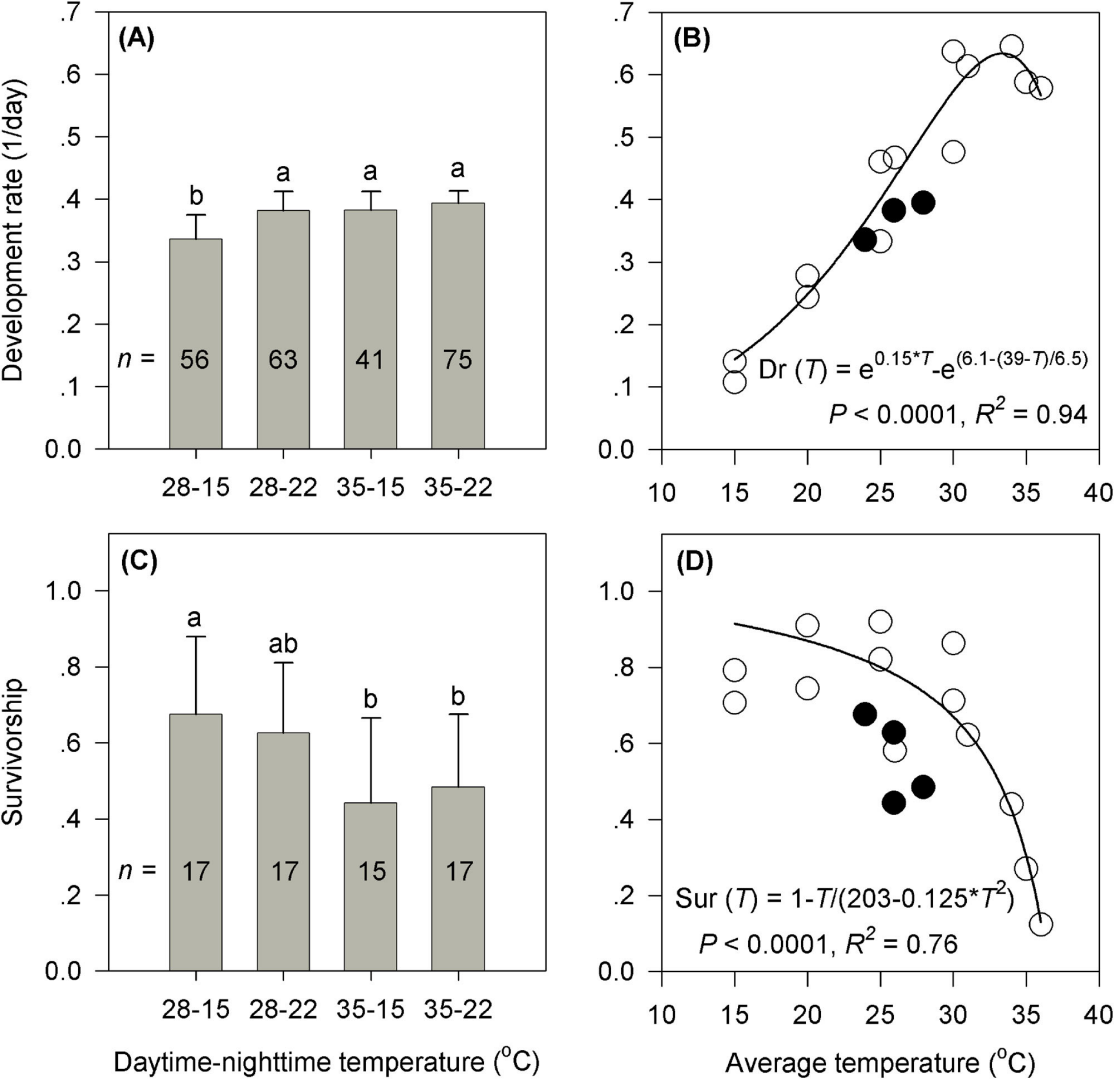
**Development rate and survivorship** **under different treatments and predictions based on daily average temperatures and observed results.** (A,C) Development rate and hatching rate of eggs under different combinations of daytime maximum and night-time minimum temperatures. The number inside each bar indicates the sample size (*n*) for each treatment. Different letters represent a significant difference between treatments (*P*<0.05). (B,D) Nonlinear models describing development rate and hatching rate of eggs based on daily average temperatures. Solid lines represent models based on various constant temperatures. Open and solid circles represent observations under constant temperatures (data from Cheng et al., 2007; Gao et al., 2007; Chen et al., 2009) and our fluctuating temperatures, respectively.

The development rate of eggs differed significantly between treatments (*F*_3,234_=46.65, *P*<0.0001). The development rate (0.335±0.038; mean±s.d.) was slower under the treatment with both lower daytime and night-time temperatures (28°C–15°C), while development rate varied between 0.382 and 0.394 under other treatments (Fig. 1A). The developmental rate at constant temperatures estimated from the literature (Cheng et al., 2007; Gao et al., 2007; Chen et al., 2009) provided a good fit to the Lactin model (Lactin et al., 1995) (Fig. 1B). Based on this model, the egg development rate was predicted to rise in an approximately linear manner with the average temperatures (24.0°C∼28.0°C) in our treatments, instead of an evident deceleration under the treatment with both higher daytime and night-time temperatures (35°C–22°C).

### Survival

Treatment significantly affected egg hatching rate (*F*_3,65_=5.01, *P*=0.0036). Survivorship was lower under treatments with a higher daytime maximum temperature of 35°C (0.44∼0.48) than under the treatment with both lower maximum and minimum temperatures (0.68±0.20) (28°C–15°C) (Fig. 1C). Egg survival under constant temperatures (Cheng et al., 2007; Gao et al., 2007; Chen et al., 2009) fitted a nonlinear model (Fig. 1D). Based on this relationship, egg survival was expected to decrease smoothly with the average temperatures rather than dramatically decline under the treatments with a daytime maximum temperature of 35°C.

### CT_max_

**Fig. 2.**
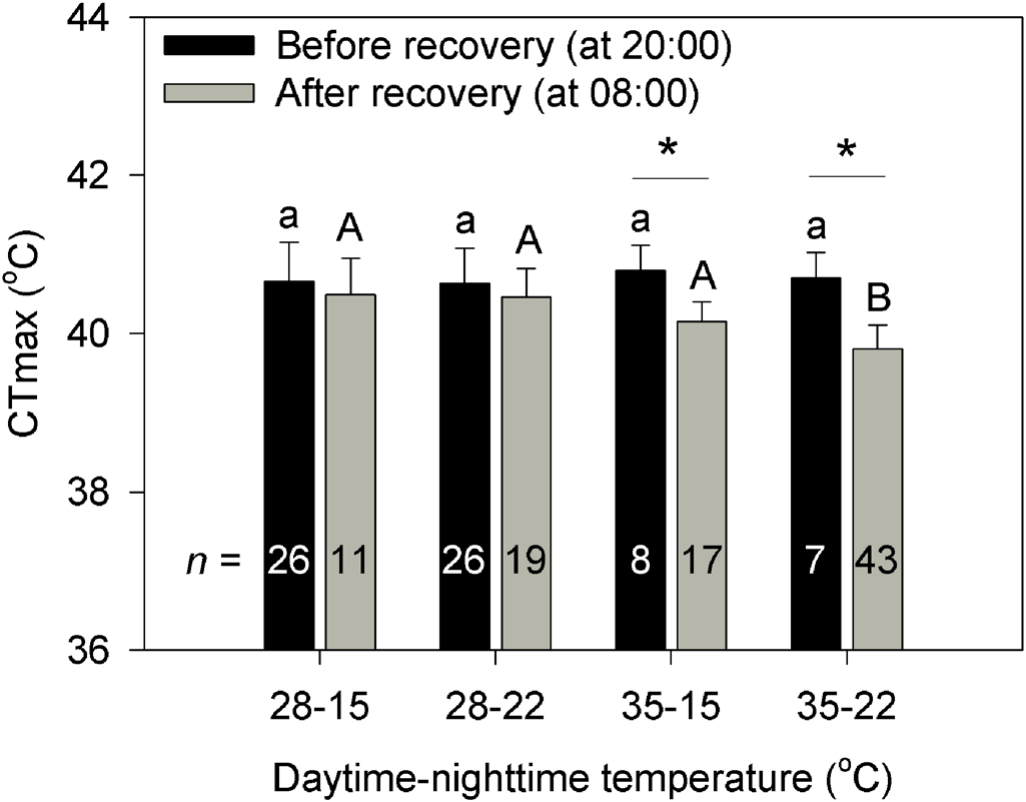
**CT_max_ of first-instar larva at 20:00 h before recovery or at 08:00 h after recovery under different combinations of daytime maximum and night-time minimum temperatures.** The number inside each bar indicates the sample size (*n*) for each treatment. Different letters represent significant differences between treatments when measuring before or after recovery at *P*=0.05. Asterisks show significant differences between measurements for CT_max_ before and after recovery within the same treatment.

The time at which the CT_max_ was measured (*F*_1,156_=33.36, *P*<0.0001) significantly affected the CT_max_ of first-instar larvae, but different thermal treatments had little effect (*F*_3,156_=0.19, *P*=0.90). However, the interaction between the time the CT_max_ was measured and temperature treatments significantly affected the CT_max_ (*F*_3,156_=6.40, *P*=0.0004). When measured at 20:00 h (i.e. before night-time recovery), CT_max_ did not significantly differ between treatments (*F*_3,89_=0.62, *P*=0.60) (Fig. 2). However, when measured at 08:00 h (i.e. after night-time recovery), CT_max_ was dramatically lower under the treatment with both higher maximum and minimum temperatures than under other treatments (*F*_3,66_=6.93, *P*=0.0004). Under the treatments with lower maximum temperatures, CT_max_ did not differ significantly with the time of measurement (i.e. with measurement time before or after night-time recovery, 28°C–15°C: *F*_1,36_=0.98, *P*=0.33; 28°C–22°C: *F*_1,44_=2.06, *P*=0.15). However, under the treatments with higher maximum temperatures, the values of CT_max_ measured after night-time recovery were much lower than those measured before recovery (35°C–15°C: *F*_1,24_=25.15, *P*<0.0001; 35°C–22°C: *F*_1,49_=47.32, *P*<0.0001).

**Fig. 3.**
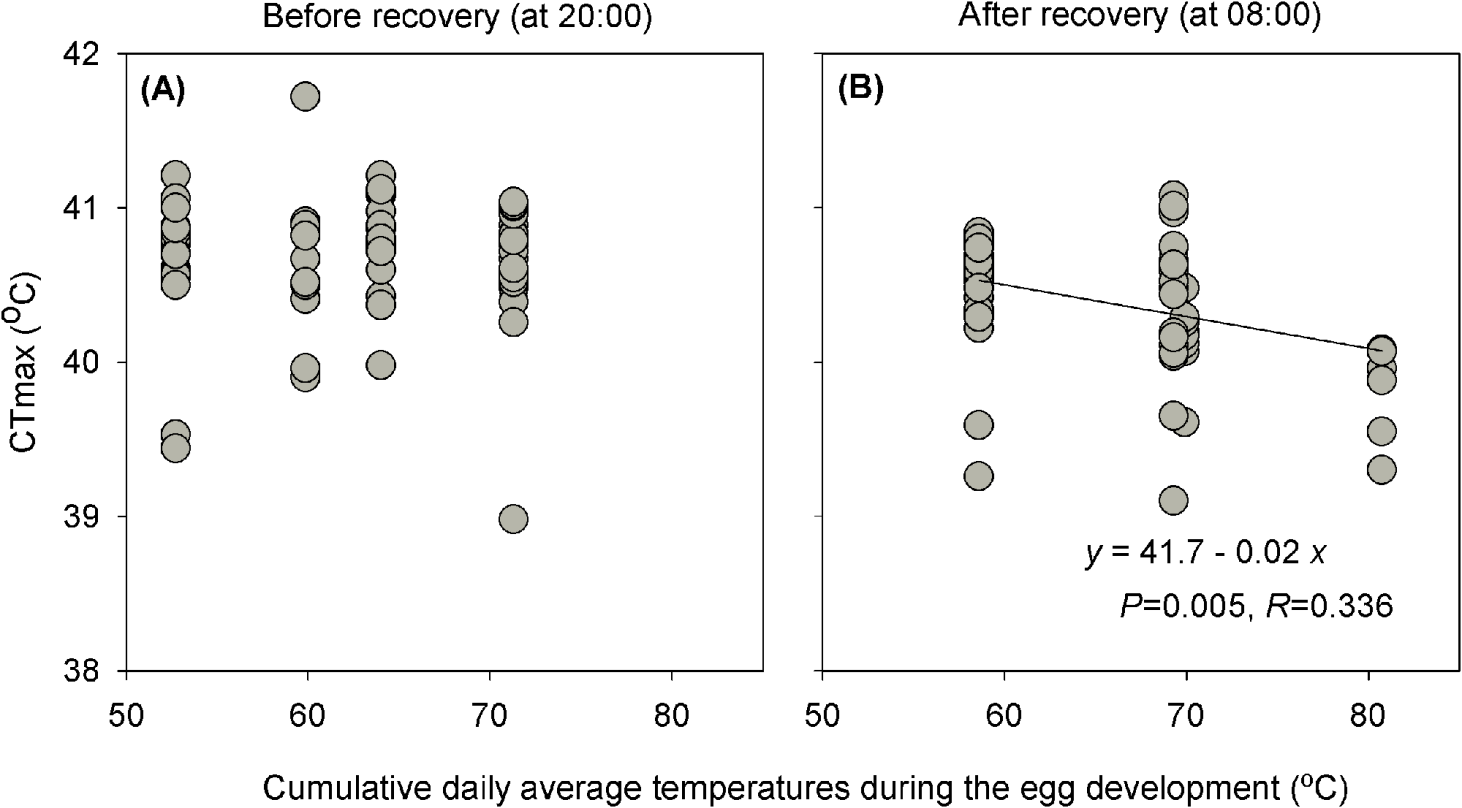
**Relationship between CT_max_ of first-instar larva and cumulative daily average temperatures during the egg development for different treatments.** (A) CT_max_ measured at 20:00 h before recovery. (B) CT_max_ measured at 08:00 h after recovery. The sample size (*n*) for each treatment is the same as in Fig. 2.

Before night-time recovery, there is no obvious correlation between CT_max_ of first-instar larvae and cumulative daily average temperatures during the egg development for different treatments (Fig. 3A). However, CT_max_ measured after recovery decreased linearly with cumulative daily average temperatures of different combinations of daytime heat stress and night-time recovery (Fig. 3B).

## DISCUSSION

Climate warming leads to changes in the means and variability of temperature (Easterling et al., 1997; IPCC, 2013). Despite recent progress on the consequences of increased temperature variability on organismal performance (Paaijmans et al., 2010; Lambrechts et al., 2011; Bozinovic et al., 2013; Xing et al., 2014), the independent and combined effects of daytime heat stress and night-time recovery remain unclear. We compared the thermal effects of daytime heat stress and night-time recovery on organismal performance in a lady beetle species. Thermal effects differed between daytime heat stress and night-time recovery, and the extent of recovery was of great importance for predicting the daily changes in organismal performance. The combined effects of daytime heat stress and night-time recovery determined thermal performances. Our findings thus provide an insight into the thermal effect of day-to-night fluctuating temperatures and have important implications for predicting the impacts of daytime and night-time warming under climate change.

### Differences in thermal effects between daytime and night-time temperatures

Thermal effects differ between daytime and night-time temperatures. High daytime temperatures had negative effects and night-time mild temperatures had positive effects. Most organisms experience fluctuating ambient temperatures during their daily life activities (Fischer et al., 2011; Colinet et al., 2015). On the one hand, organisms often encounter diurnal high temperatures beyond their sublethal thermal maxima which may force them to endure a period of heat stress (Ma et al., 2015a; Bozinovic et al., 2016; Stoks et al., 2017). We found differences in egg survival across temperature regimes. Hatching rate was lower in eggs that experienced temperature fluctuations that included a higher daytime maximum temperature (Fig. 1C). Furthermore, compared to predictions based on various constant-temperature models, egg survival was markedly reduced under the regimes that had the same average temperature but a daytime maximum temperature of 35°C (Fig. 1D). These results suggest that daytime heat stress had adverse impacts on egg survival.

While organisms may experience adverse high temperatures during the day, they may also experience mild nocturnal temperatures that may provide recovery opportunities to repair heat injuries (Bozinovic et al., 2011; Colinet et al., 2015; Speights et al., 2017). When experiencing a higher daytime maximum temperature of 35°C, first-instar larvae had a lower CT_max_ after night-time recovery than before recovery (Fig. 2). The result indicates that the larvae show a rapid hardening after undergoing daytime high temperatures to enhance their heat tolerance. The increase in heat tolerance, however, is generally at the cost of increased production of heat shock proteins (HSP) which may be associated with decreases in some important life-history traits such as development and reproduction (Feder and Hofmann, 1999; Sørensen et al., 2003; McMillan et al., 2005). In contrast, after experiencing night-time mild temperatures, larvae may recover and return to a normal level of heat tolerance. Organisms can compensate for development and reproduction at moderate thermal environments by decomposing HSPs, which can prevent them from suffering heat injuries (Feder and Hofmann, 1999; McMillan et al., 2005). The fact that heat shock survival of the fruit fly *Drosophila melanogaster* shows a significant positive association with daily fluctuating temperatures (Overgaard and Sørensen, 2008) also implies different thermal effects between daytime heat stress and night-time recovery.

### Whether or not and the extent of night-time recovery matters in predicting daily thermal effects

Whether or not organisms recover at moderate temperatures is important for predicting daily thermal effects. After stressed with a daytime maximum temperature of 35°C, the larvae had higher CT_max_ without a recovery period relative to those with a recovery period (Fig. 2). This result implies that the effect of night-time recovery on thermal performance, such as heat tolerance, is counteracted by rapid heat hardening during high daytime temperatures. Our findings, to some extent, can also be supported by the evidence that organisms have lower survival and fecundity when extreme high temperature events or hot days occur more frequently (Ma et al., 2004a,b; Gillespie et al., 2012; Sentis et al., 2013; Ma et al., 2015a). Mild temperatures play an important role in recovering and repairing from multiple heat waves (Bozinovic et al., 2011; Zhao et al., 2014; Ma et al., 2018).

Night-time temperatures determine the extent to which organisms may recover from daytime heat stress. Based on the thermal performance curves and the ‘Kaufmann effect’, development rate is predicted to rise in an approximately linear manner with temperature within a narrow range of temperatures for both constant or fluctuating conditions when average temperature is below the optimum temperature (Worner, 1992; Ragland and Kingsolver, 2008; Bozinovic et al., 2011; Estay et al., 2014). Based on this prediction, egg development rate was hypothesized to increase approximately linear with average temperature in our four treatments. However, development rate under the regime of 35–22°C equaled that of the 35–15°C regime, indicating that even though the average temperature was higher for that first treatment, egg development did not increase as expected, which suggests that the expected increase was dampened by the regime with higher daily temperature minima (Fig. 1A,B). In addition, when experiencing the same daytime heat stress with a daytime maximum temperature of 35°C, the CT_max_ differed significantly between the larvae recovering at night-time minimum temperatures of 22°C and 15°C (Fig. 2). These results suggest that the extent of night-time recovery may lead to different effects on thermal performance, such as heat tolerance. When experiencing similar daytime temperature fluctuations (Zhao et al., 2014), adult aphids of *Sitobion avenae* produced more offspring at relatively lower night-time temperatures (minima varied between 13°C–19°C) than those at higher night-time temperatures (minima varied between 21°C–25°C), which also indicates that night-time temperatures affected the different degrees of recovery from heat stress during the day. The extent to which organisms recover at moderate thermal conditions from multiple instances of heat stress may cause changes in life-history traits and lead to consequences at the population level (Ma et al., 2018).

### Combined effects of daytime and night-time temperatures determine performance

The negative effects of stressful daytime high-temperatures, combined with the positive effects of moderate night-time temperatures determine thermal performances. Increases in daytime high temperatures can cause decreased development rate, survival and reproduction and thus decrease population growth, even when the temperature at night remains suitable (Ma et al., 2015a,b). On the other hand, decreases in mild night-time temperatures may result in increased development, longevity and reproduction even if there is a stressful temperature fluctuation during the daytime (Zhao et al., 2014). These facts indicate that the extent of both daytime negative and night-time positive effects are important for modelling daily thermal effects. Here we find that the performances including development, survival and heat tolerance differed under the regimes with different combinations of heat stress and recovery (Figs 1,2). Furthermore, the CT_max_ decreases linearly with the cumulative daily average temperatures during the egg development (Fig. 3B), which also implies a correlation between thermal performance and the combined effect of daytime heat stress and night-time recovery. These results, together with previous evidence, suggest that organism thermal performance is determined by the combined effects of varying degrees of daytime heat stress and night-time recovery.

### Implication for biological consequences of daytime and night-time warming

Climate change has resulted in a greater increase in daily minima than in maxima (Karl et al., 1993; Easterling et al., 1997). The asymmetric shifts in diurnal fluctuating temperatures lead to a more marked night-time warming than daytime warming (IPCC, 2013). Night-time warming is thus expected to be more important for organisms (Clarke and Zani, 2012; Zhao et al., 2014; Barton and Schmitz, 2018). Warmer nights will likely prevent organisms from recovering from daytime heat stress (Colinet et al., 2015; Zhao et al., 2014; Ma et al., 2018). On the other hand, according to thermal performance curves, even a small increase in temperatures above thermal maxima can cause a dramatic decline in individual performance (Angilletta, 2009; Bozinovic et al., 2011). Therefore, the influence of daytime warming is also of significance in individual performance (Gillespie et al., 2012; Sentis et al., 2013; Ma et al., 2015a,b; Stoks et al., 2017). The negative impacts of daytime heat stress may be aggravated by higher temperatures in hotter days (Ma et al., 2015a,b; Barton and Schmitz, 2018). Given that heat tolerance is found to be evolutionarily conserved and organisms are expected to have limited plasticity to increase their upper thermal limits (Terblanche and Chown, 2006; Potter and Woods, 2012; Hoffmann et al., 2013; Heerwaarden et al., 2016; Kellermann et al., 2017), both daytime and night-time warming due to climate change are likely to result in substantial changes in thermal performance and thus may cause severe ecological consequences.

## MATERIALS AND METHODS

### Study insects

We studied beetles (*P. japonica*) that were 8th to 10th generation descendants of beetles that were originally collected from a corn field at Langfang Experimental Station (39.51°N, 116.61°E) of the Institute of Plant Protection, Chinese Academy of Agricultural Sciences in September 2013. The beetles were maintained in screen cages (60×60×60 cm) in a rearing room with constant temperature 22±1°C and relative humidity 60–70%, and a photoperiod of 16:8 (L:D). The larvae and adults were fed with the English grain aphid (*S. avenae*) feeding on 5–10 cm high winter wheat seedlings. The beetle eggs were collected and then put in petri dishes (diameter: 5 cm; height: 1 cm) with moist filter papers to allow them to hatch. After hatching, the first-instar larvae were transferred to the screen cages and fed with the grain aphids. Larvae, pupae and adults were transferred to new cages weekly.

### Experiment design

**Fig. 4.**
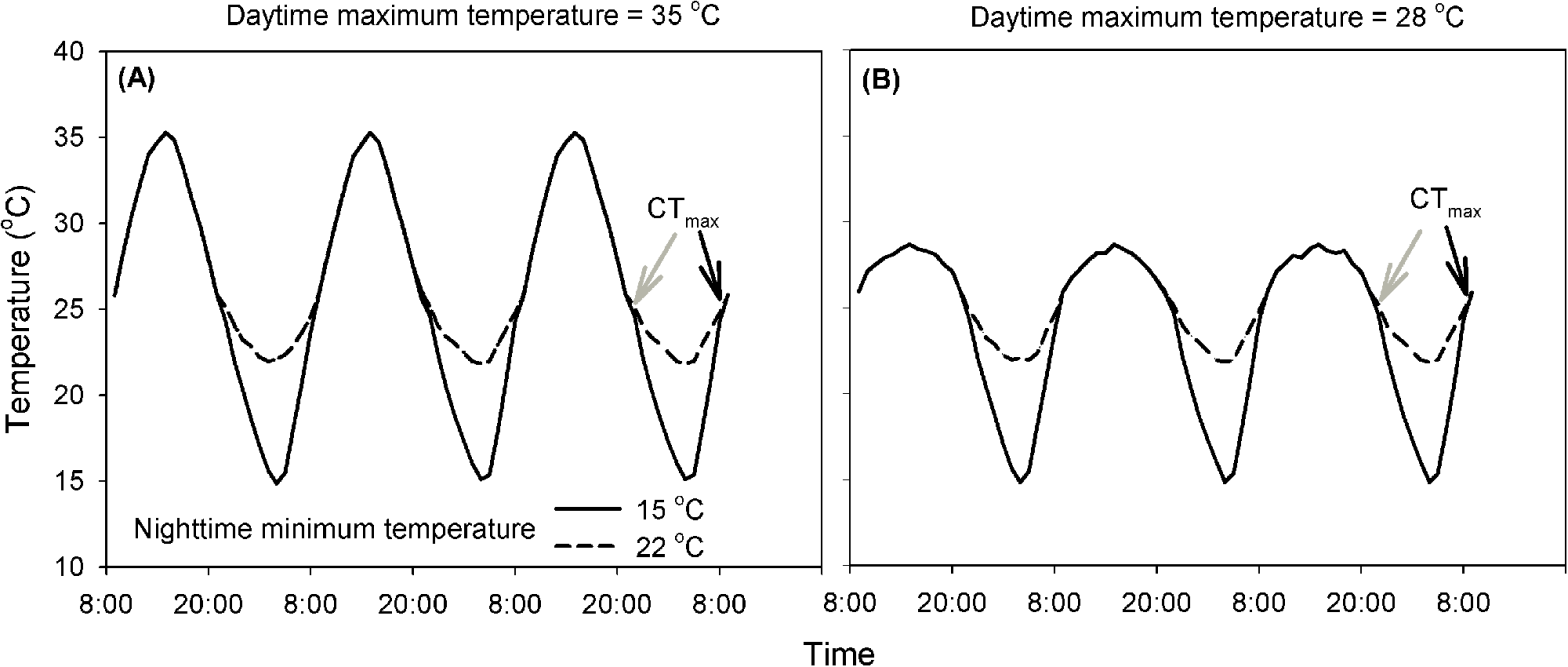
**Temperature regimes used for creating different combinations of daytime heat stress and night-time recovery.** (A) Temperature regimes with different night-time minimum temperatures and a higher daytime maximum temperature of 35°C. (B) Temperature regimes with different night-time minimum temperatures and a moderate daytime maximum temperature of 28°C. Grey and black arrows represent the time for measuring CT_max_ before and after night-time recovery, respectively.

We created a factorial design to differentiate between the thermal effects of daytime heat stress and night-time recovery. We independently manipulated the daytime maximum and night-time minimum temperatures to test how changes in daytime and night-time temperatures affected development, survival and CT_max_. The temperature regimes (maximum-minimum temperature) were: 28°C–15°C (moderate stress and strong recovery), 28°C–22°C (moderate stress and moderate recovery), 35°C–15°C (strong stress and strong recovery) and 35°C–22°C (strong stress and moderate recovery), with daily average temperatures of 24, 26, 26 and 28°C, respectively (Fig. 4A,B). Since the optimal temperature for the lady beetle *P. japonica* is 25°C (Yang, 1985; Gao et al., 2007; Chen et al., 2009), the daytime maximum temperature of 28 and 35°C can be treated as moderate and strong stress respectively.

Four climate chambers (RXZ-280B, Jiangnan Ltd., Ningbo, China) were used to establish the temperature regimes. Temperature in each chamber was manipulated to change gradually as a 24-h fluctuation and was cycled during the experiment. At the beginning of the experiment, temperature started to increase at 08:00 h, reached and stayed at a high level (28 or 35°C) from 13:00–14:00 h, and then decreased to and remained at a low level (22 or 15°C) from 01:00–02:00 h. After that the temperature started to increase again. All the regimes lasted for three consecutive days during the experiment (Fig. 4A,B).

### Development and survival of eggs

To understand how changes in daytime heat stress and night-time recovery affect key fitness components, the development and survival of eggs under the four temperature regimes were examined. Egg masses laid within the past 12 h were collected as test insects. The number of egg for each mass was counted. The egg masses were put in a petri dish (diameter: 7 cm, height: 1 cm) separately and then placed in the chambers. For each treatment, 18 egg masses including about 200 eggs were tested. When the experiment began, the development of eggs was checked twice per day at 08:00 h and 20:00 h. If the first-instar larvae hatched, the time and the number of hatched eggs in each petri dish were recorded. Then the newly hatched first-instar larvae were collected to test their CT_max_. The rest of eggs were returned to the chambers and were checked as before. In this experiment, all the live eggs were found to develop into first-instar larvae in 2.5 or 3 days, and the eggs that failed to hatch in three days died. The development time of each egg was used to calculate its development rate. The proportion of hatched larvae of each egg mass was calculated as hatching rate.

### CT_max_ of first-instar larvae

To reveal the effect of daytime heat stress and night-time recovery on heat tolerance, the CT_max_ of newly hatched first-instar larva was tested. Specifically, the CT_max_ of the larvae hatched at different times (at either 20:00 h without or 08:00 h with night-time recovery, i.e. at 2.5 or 3 days, respectively) (Fig. 4A,B) were compared to test whether and how the extent of night-time recovery affected thermal performance. Newly hatched first-instar larvae were collected and placed individually into a tube (diameter: 5 mm, height: 3 mm) of a honeycomb plate (length: 15 cm, width: 15 cm) with the bottom covered by nylon gauze. Then the tubes of the plate were covered with a transparent plastic sheet (length: 15 cm, width: 15 cm, thickness: 0.5 mm). After that, the plate with the test larvae was put into a bottle (inner diameter: 16 cm, height: 18 cm) that was heated by the circulating liquid (glycol), filled between the inner and outer walls. The rate of temperature increase was regulated by a refrigerated heating circulator bath (Julabo F34-HE; JULABO GmbH Ltd., Germany; accuracy: 0.01°C). The bath was connected to a computer to run the program of temperature increase and record the temperature and time at which the test larvae ceased walking and started to twitch (i.e. the CT_max_). Test larvae were allowed to settle for 15 min prior to measurement. First they were heated at 0.25°C min^−1^ from 25°C to 31°C. Then they were heated at 0.1°C min^−1^ from 31°C to 42°C. During this time, a digital video camera was used to record the behavior of the larvae. The 0.1°C min^−1^ rate was selected because it was analogous to field conditions and thus was ecologically meaningful (Chown et al., 2009; Hazell et al., 2010; Ma and Ma, 2012). In a preliminary study, all the first-instar larvae died when the temperature reached 42°C. Hence the CT_max_ test stopped at this temperature. Here we did not consider the possibility of non-random mortality of the larvae during temperature treatments on the results of the CT_max_ trials.

### Statistical analysis

We analyzed the effects of treatments on the development rate and hatching rate of eggs using a generalized linear model (GLM) with GENMOD procedure in SAS Version 8 followed by planned contrasts based on least-square means to compare the levels of significant differences between treatments. We analyzed the effect of treatment and measuring time on CT_max_ of first-instar larva using two-way ANOVA and normally distributed errors using the GLM procedure, and the means were separated with Duncan's multiple range tests (*P*<0.05). To indicate how CT_max_ of first-instar larvae is linked to changes in degrees of the combination of heat stress and recovery, the relationship between CT_max_ and cumulative daily average temperature during the egg development was established through linear regression in SAS Version 8.
